# Supplementary material for: Posture similarity index: a method to compare hand postures in synergy space
Source: PeerJ. 2018 Dec 10;6:e6078. doi: 10.7717/peerj.6078 (PMC6292379; doi:10.7717/peerj.6078)

Internally constrained hand postures

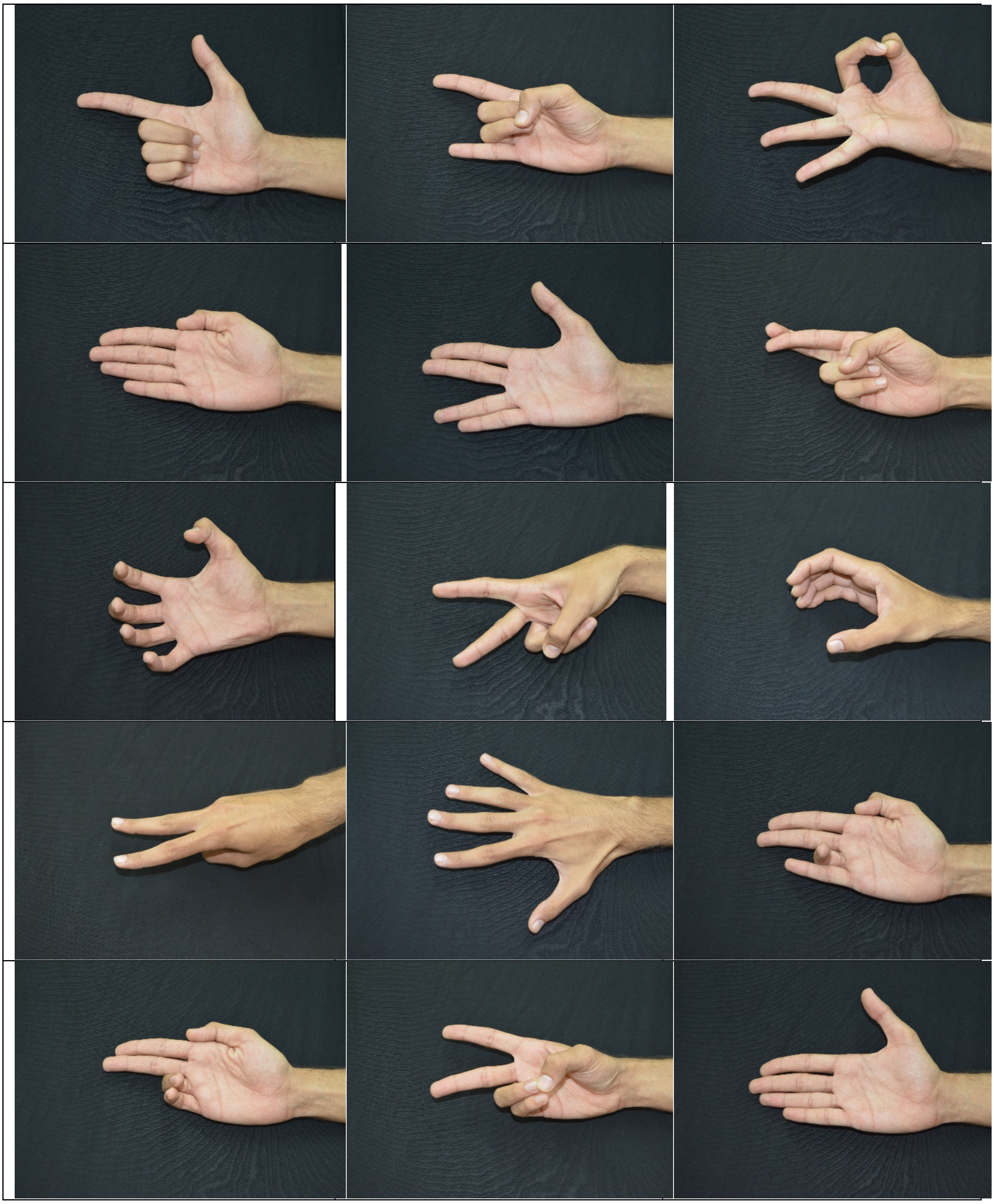

Continued

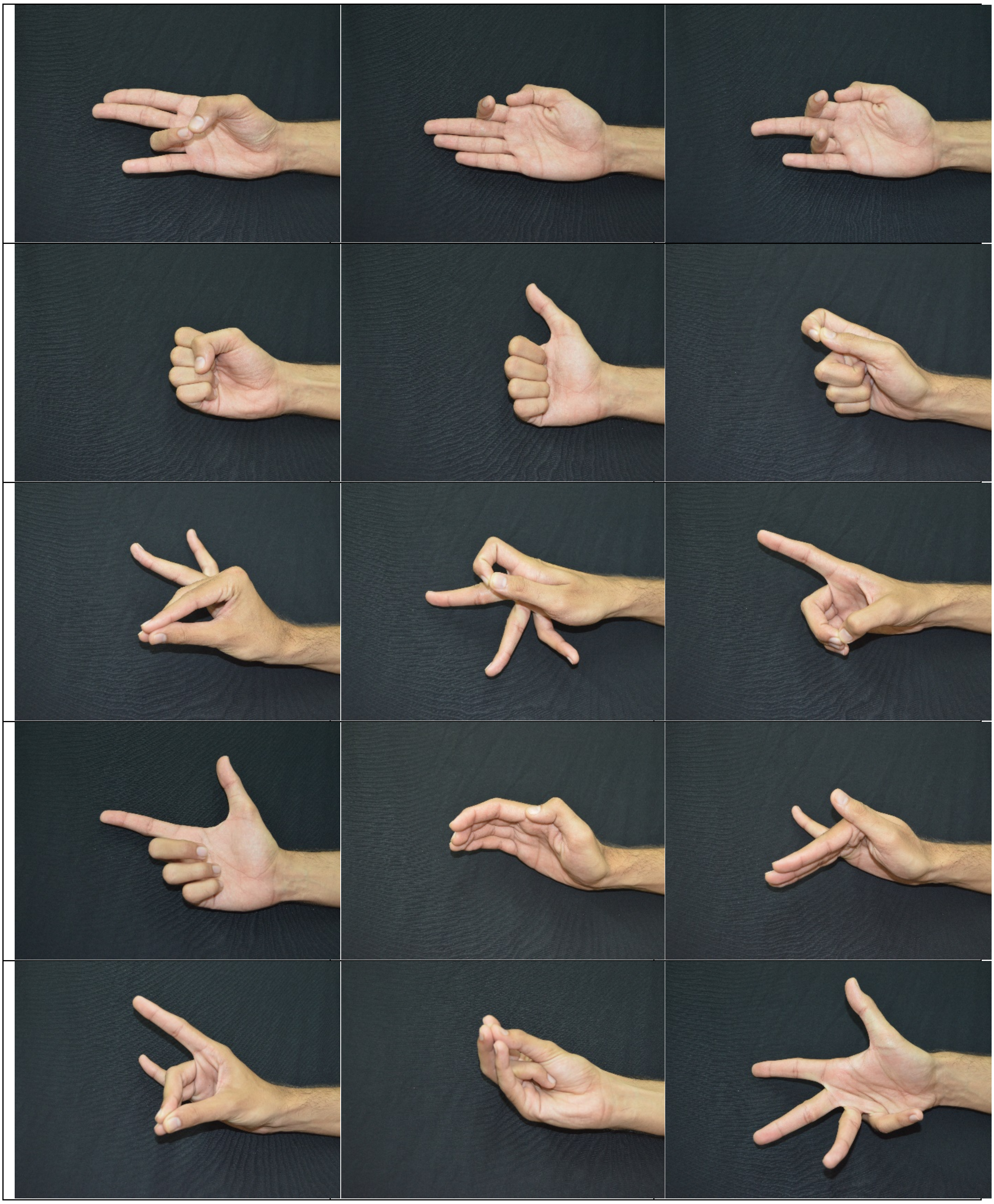

Continued

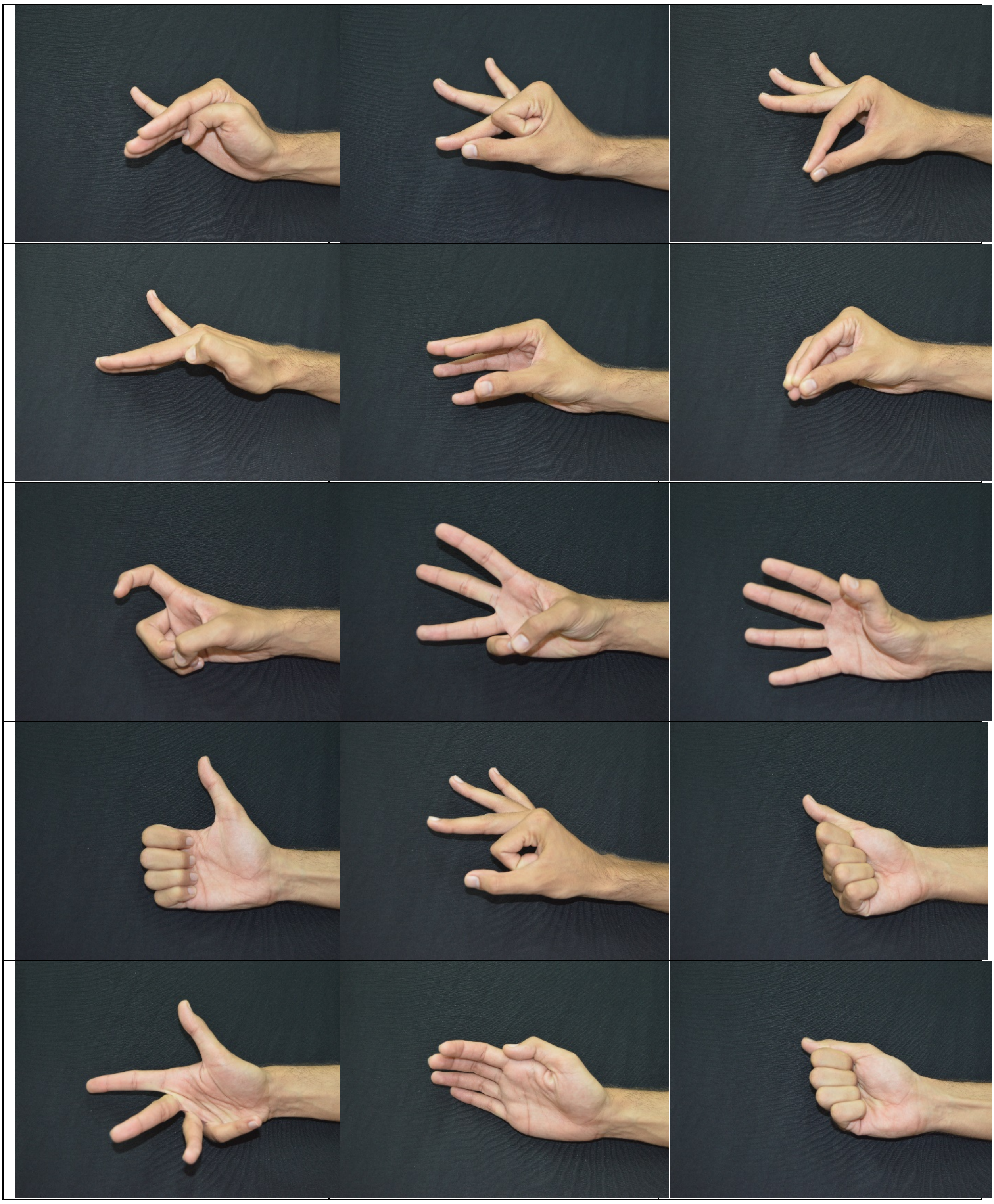

Continued

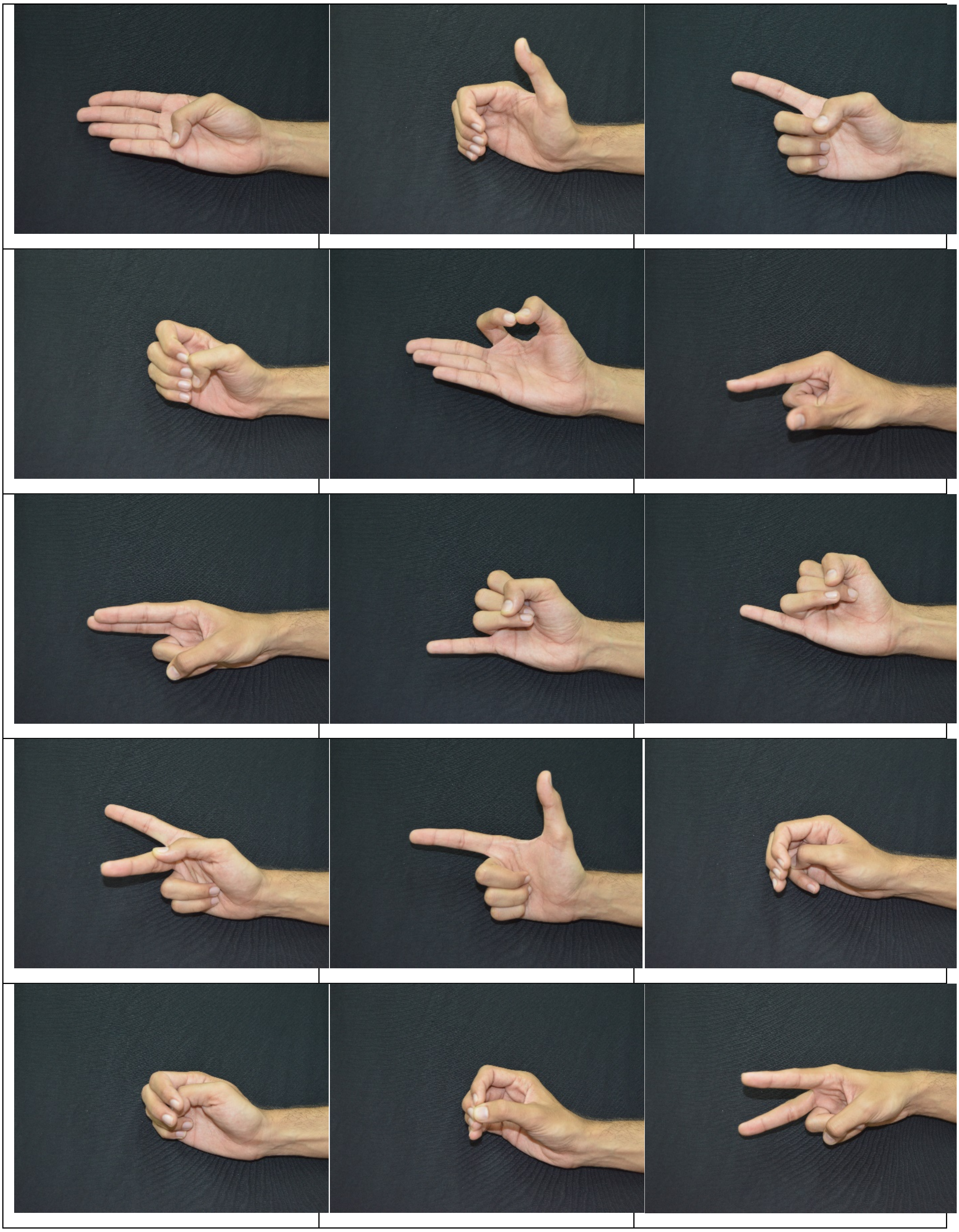

Continued

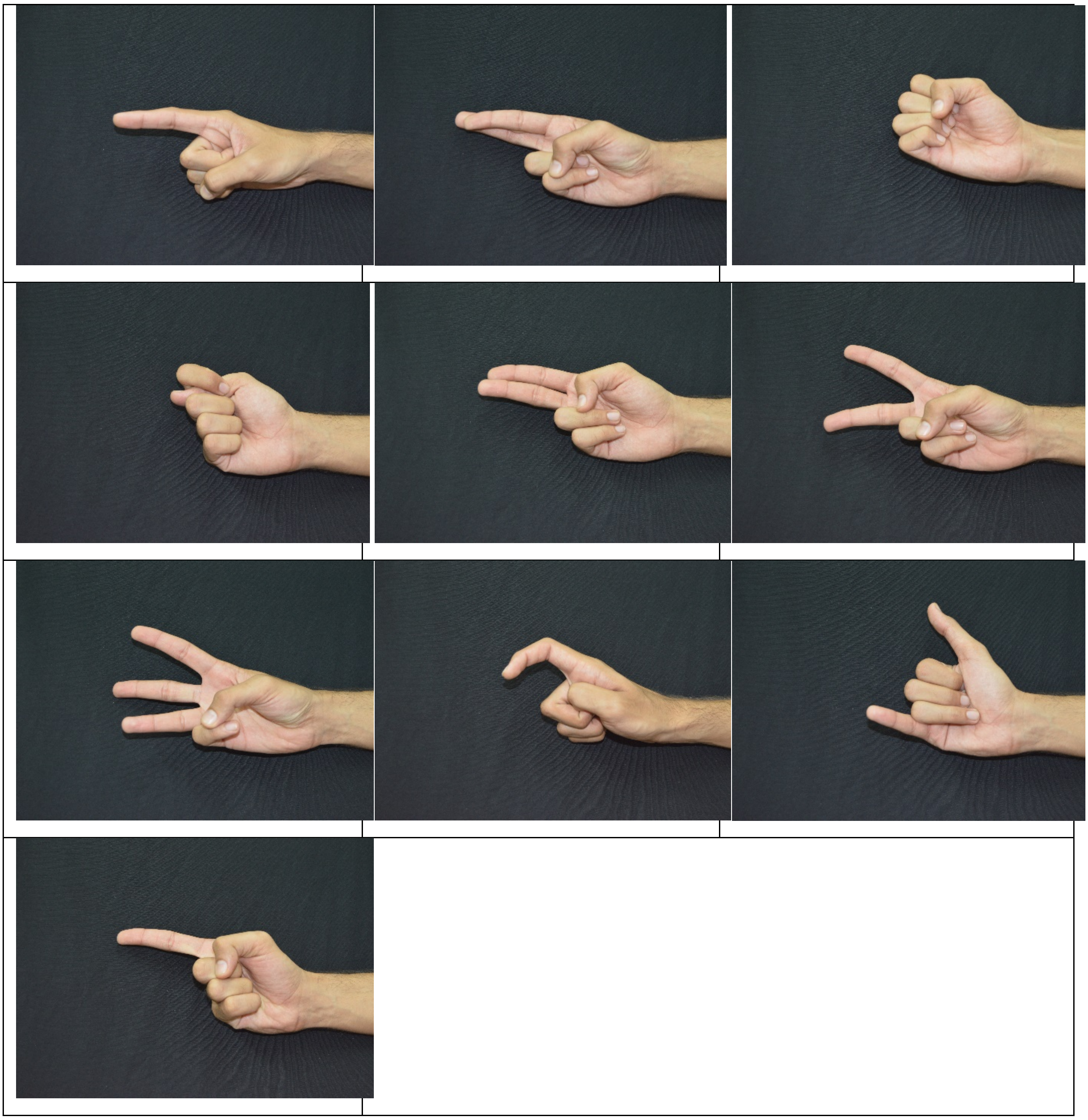

Externally constrained hand postures:

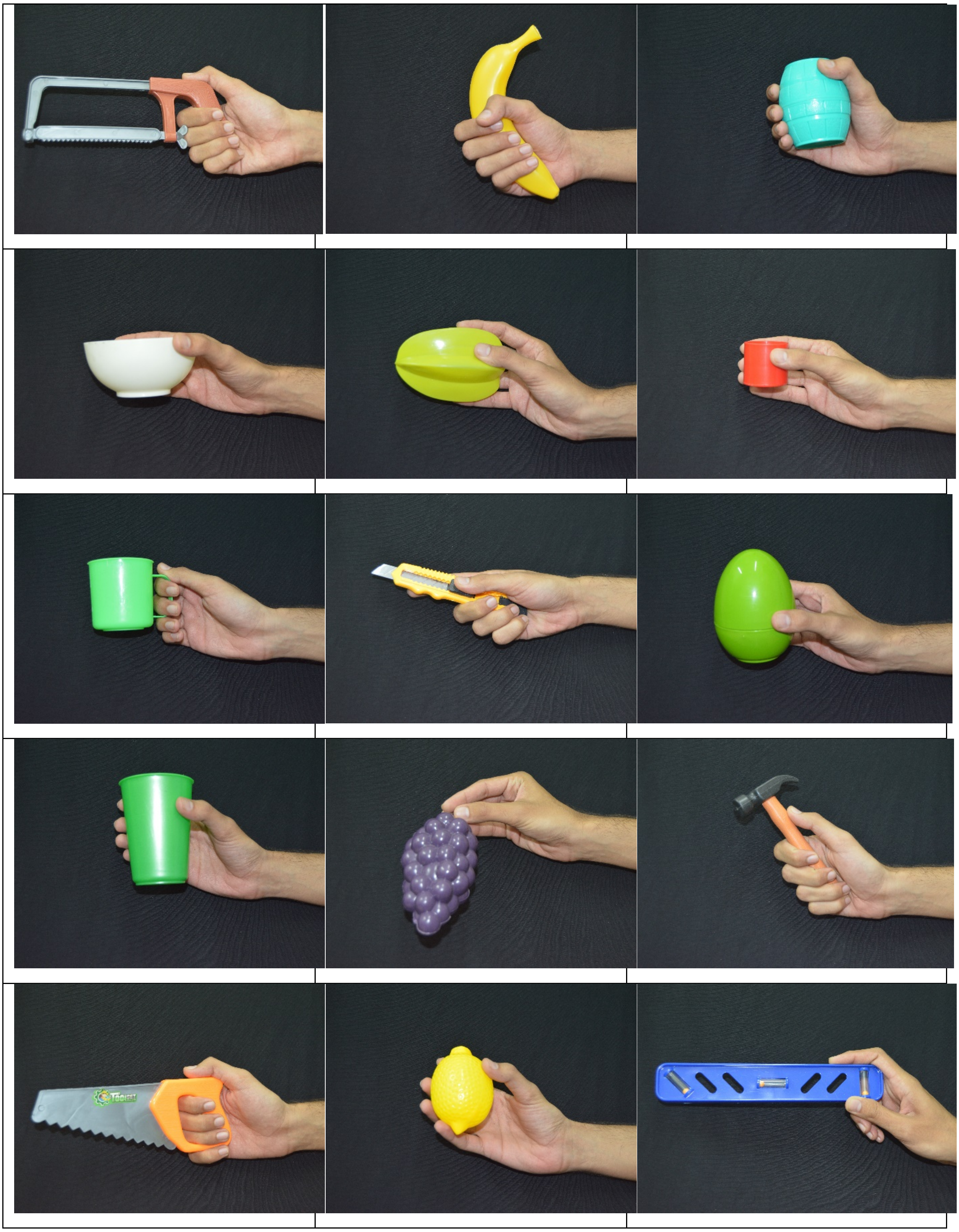

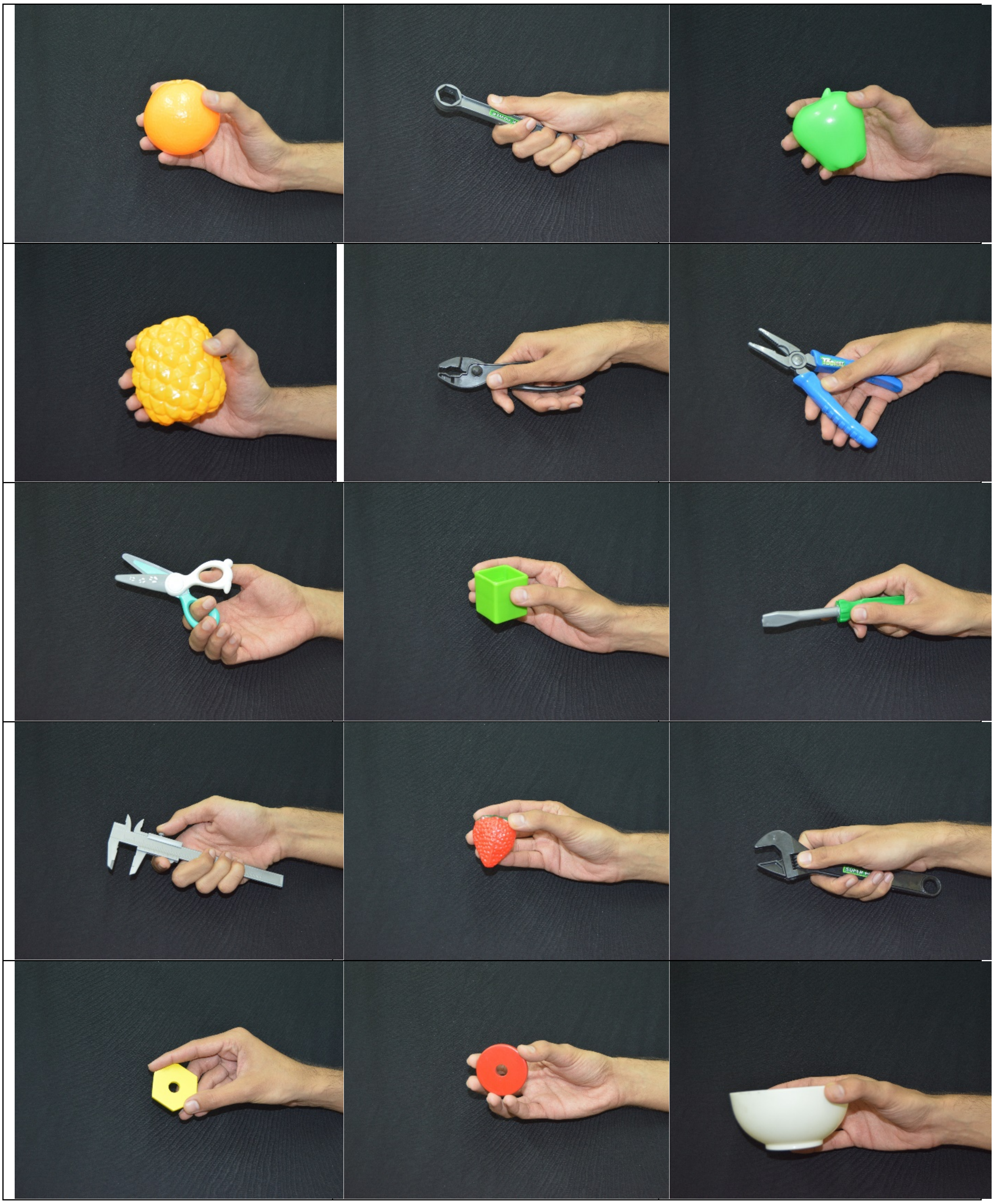

Supplement: Supplemental Information 2 — A list of all hand postures used in this experiment. All photographs by Varadhan SKM. Hand model: Nayan Bhatt. [file peerj-06-6078-s002.pdf]
