# Supplementary material for: Posture similarity index: a method to compare hand postures in synergy space
Source: PeerJ. 2018 Dec 10;6:e6078. doi: 10.7717/peerj.6078 (PMC6292379; doi:10.7717/peerj.6078)
Supplement: Supplemental Information 3 [file peerj-06-6078-s003.pdf]

Table S1: Ratio of variances in each PC directions. Superscript (i,j) represents the ratio of variances of ith and j[th] posture. Subscript (n) represents n[th] principal component

${F_1}^{1,1} = 1$	${F_1}^{1,2} = 1.0476$	${F_1}^{1,3} = 0.9565$
${F_1}^{2,1} = 0.9565$	${F_1}^{2,2} = 1$	${F_1}^{2,3} = 0.9130$
${F_1}^{3,1} = 1.0455$	${F_1}^{3,2} = 1.0952$	${F_1}^{3,3} = 1$
${F_2}^{1,1} = 1$	${F_2}^{1,2} = 1.1034$	${F_2}^{1,3} = 267.8606$
${F_2}^{2,1} = 0.9063$	${F_2}^{2,2} = 1$	${F_2}^{2,3} = 242.7487$
${F_2}^{3,1} = 0.0037$	${F_2}^{3,2} = 0.0041$	${F_2}^{3,3} = 1$
${F_3}^{1,1} = 1$	${F_3}^{1,2} = 1$	${F_3}^{1,3} = 24.0044$
${F_3}^{2,1} = 1$	${F_3}^{2,2} = 1$	${F_3}^{2,3} = 24.0044$
${F_3}^{3,1} = 0.0417$	${F_3}^{3,2} = 0.0417$	${F_3}^{3,3} = 1$

Table S2: PSI matrix for synthetic dataset

$\boldsymbol{PSI_{1,1}} = 0.062 * 1 + 0.0127 * 1 + 0.0054 * 1 = 0.0800$	$\boldsymbol{PSI_{1,2}} = 0.062 * 0.9565 + 0.0127 * 0.9063 + 0.0054 * 1 = 0.0762$	$\boldsymbol{PSI_{1,3}} = 0.062 * 0.9565 + 0.0127 * 0.0037 + 0.0054 * 0.0417 = 0.059$
$\boldsymbol{PSI_{2,1}} = 0.062 * 0.9565 + 0.0127 * 0.9063 + 0.0054 * 1 = 0.0762$	$\boldsymbol{PSI_{2,2}} = 0.062 * 1 + 0.0127 * 1 + 0.0054 * 1 = 0.0800$	$\boldsymbol{PSI_{2,3}} = 0.062 * 0.9130 + 0.0127 * 0.0041 + 0.0054 * 0.0417 = 0.056$
$\boldsymbol{PSI_{3,1}} = 0.062 * 0.9565 + 0.0127 * 0.0037 + 0.0054 * 0.0417 = 0.059$	$\boldsymbol{PSI_{3,2}} = 0.062 * 0.9130 + 0.0127 * 0.0041 + 0.0054 * 0.0417 = 0.056$	$\boldsymbol{PSI_{3,3}} = 0.062 * 1 + 0.0127 * 1 + 0.0054 * 1 = 0.0800$

Table S3: Normalized Posture Similarity Index (N PSI) for synthetic dataset

1	0.9464	0.7337
0.9464	1	0.6977
0.7337	0.6977	1

Table S4: PSI matrix for experimental test dataset

$PSI_{1,1} = 0.1152$	$PSI_{1,2} = 0.0873$	$PSI_{1,3} = 0.103$
$PSI_{2,1} = 0.0873$	$PSI_{2,2} = 0.1152$	$PSI_{2,3} = 0.0808$
$PSI_{3,1} = 0.103$	$PSI_{3,2} = 0.0808$	$PSI_{3,3} = 0.1152$

Table S5: Normalized PSI matrix for experimental test dataset

$PSI_{1,1} = 1$	$PSI_{1,2} = 0.7573$	$PSI_{1,3} = 0.8934$
$PSI_{2,1} = 0.7573$	$PSI_{2,2} = 1$	$PSI_{2,3} = 0.7012$
$PSI_{3,1} = 0.8934$	$PSI_{3,2} = 0.7012$	$PSI_{3,3} = 1$
